# Disruption of the Autophagy-Lysosome Pathway Is Involved in Neuropathology of the *nclf* Mouse Model of Neuronal Ceroid Lipofuscinosis

**DOI:** 10.1371/journal.pone.0035493

**Published:** 2012-04-20

**Authors:** Melanie Thelen, Markus Daμμe, Michaela Schweizer, Christian Hagel, Andrew M.S. Wong, Jonathan D. Cooper, Thomas Braulke, Giovanna Galliciotti

**Affiliations:** Department of Biochemistry, Children's Hospital, University Medical Center Hamburg-Eppendorf, Hamburg, Germany; Department of Biochemistry 1, University Bielefeld, Bielefeld, Germany; Center for Molecular Neurobiology, University Medical Center Hamburg-Eppendorf, Hamburg, Germany; Institute of Neuropathology, University Medical Center Hamburg-Eppendorf, Hamburg, Germany; Department of Neuroscience and Centre for the Cellular Basis of Behaviour, MRC Centre for Neurodegeneration Research, Kinǵs College London, Institute of Psychiatry, London, United Kingdom; Institute of Molecular and Cell Biology, Singapore

## Abstract

Variant late-infantile neuronal ceroid lipofuscinosis, a fatal lysosomal storage disorder accompanied by regional atrophy and pronounced neuron loss in the brain, is caused by mutations in the *CLN6* gene. CLN6 is a non-glycosylated endoplasmic reticulum (ER)-resident membrane protein of unknown function. To investigate mechanisms contributing to neurodegeneration in CLN6 disease we examined the *nclf* mouse, a naturally occurring model of the human CLN6 disease. Prominent autofluorescent and electron-dense lysosomal storage material was found in cerebellar Purkinje cells, thalamus, hippocampus, olfactory bulb and in cortical layer II to V. Another prominent early feature of *nclf* pathogenesis was the localized astrocytosis that was evident in many brain regions and the more widespread microgliosis. Expression analysis of mutant Cln6 found in *nclf* mice demonstrated synthesis of a truncated protein with a reduced half-life. Whereas the rapid degradation of the mutant Cln6 protein can be inhibited by proteasomal inhibitors, there was no evidence for ER stress or activation of the unfolded protein response in various brain areas during postnatal development. Age-dependent increases in LC3-II, ubiquitinated proteins, and neuronal p62-positive aggregates were observed, indicating a disruption of the autophagy-lysosome degradation pathway of proteins in brains of *nclf* mice, most likely due to defective fusion between autophagosomes and lysosomes. These data suggest that proteasomal degradation of mutant Cln6 is sufficient to prevent the accumulation of misfolded Cln6 protein, whereas lysosomal dysfunction impairs constitutive autophagy promoting neurodegeneration.

## Introduction

Neuronal ceroid lipofuscinoses (NCL) are a group of severe neurodegenerative lysosomal storage disorders, and are considered the most common progressive encephalopathies of childhood [1]. The NCLs are caused by mutations in at least nine human CLN genes, which can be linked to the onset and clinical course of the disease [1], [2]. The different forms share common clinical features, including progressive loss of vision, mental and motor deterioration, epileptic seizures, and premature death [3]. These disorders are characterized by accumulation of autofluorescent ceroid lipopigments in most tissues, but pathological effects are most pronounced in the central nervous system, which displays a progressive and remarkably selective loss of neurons [4], [5]. The *CLN6* gene encodes a polytopic non-glycosylated membrane protein of unknown function. CLN6 is localized in the ER [6], [7] and widely expressed in many tissues [8], [9]. In the mouse brain, high *Cln6* mRNA levels are found in diverse neuronal populations, in layer II-VI of the cerebral cortex, the hippocampal CA1 and dentate gyrus regions, and in the cerebellar Purkinje cell layer [10]. CLN6 is conserved across vertebrates showing no sequence homology with other proteins. Mutations in *CLN6* cause variant late-onset neuronal ceroid lipofuscinosis (vLINCL [8], [9]) as well as an adult form of the disease [11]. Among the 55 disease-causing mutations identified in the *CLN6* gene (http://www.ucl.ac.uk/ncl/cln6.shtml), a 1-bp insertion in exon 4 (c.316insC) causes a frame shift and a premature stop codon [12] leading to the production of a truncated protein (p.R106PfsX26). In the mouse ortholog, an identical mutation (c.307insC) has been identified in *nclf* mice [7], [8], a naturally occurring model for CLN6 disease recapitulating the phenotype of the disease [13]. Homozygous *nclf* mice develop progressive retinal atrophy early in life, show cerebral atrophy, spastic limb paresis at eight months, paralysis and premature death at one year of age.

Little is known about the mechanisms leading to neurodegeneration in any form of NCL. Whereas the accumulation of storage material increases linearly with age, in some brain regions a more rapid progression of the disease has been observed [14]. Indeed, the rate and distribution of accumulating lipofuscin-like lipopigments correlate only partially with the severity of the neuron loss, and not all cells exhibiting increased storage material go on to die [15]. It is also apparent that this stored material is complex in nature with accumulation of the c-subunit of mitochondrial ATP synthase or saposins, and secondary accumulating lipids such as glycosphingolipids, cholesterol, dolichol, ubiquinone or bis(monoacylglycero)phosphate [16], [17]. Rather than storage material accumulation, localized activation of glia is more closely related to the distribution of subsequent neuron loss [14], [18], [19]. Thus, sites showing early reactive changes display the most pronounced neuron loss, with selective loss of different subtypes of GABAergic interneurons in these brain regions [14], [20].

ER stress activation due to the accumulation of misfolded proteins leads to unfolded protein response (UPR) and has already been reported in several neurodegenerative disorders, including some forms of NCL [21], [22]. A consequence of UPR is the activation of the ubiquitin-proteasome system, which is responsible for degradation of misfolded proteins or small aggregates [23], [24]. In addition, the constitutive autophagy-lysosome pathway serves the degradation of long-lived proteins, damaged organelles, clearance of toxic protein aggregates and intracellular pathogens [25]. Recent studies showed that loss of autophagy results in cytoplasmic accumulation of ubiquitin-positive aggregates and neurodegeneration [26], [27]. For CLN3 and CLN10, a disturbance of autophagy has already been shown [28], [29]. Both proteasomal and autophagic systems are indispensable for maintaining cellular homeostasis by working in a coordinated way in removing some abnormal proteins [30].

In this study, we have investigated several mechanisms such as proteasomal degradation, ER stress, autophagy and reactive gliosis, which might contribute to neurodegeneration in CLN6 disease using *nclf* mice as a model system.

## Results

### Gross morphology of the central nervous system and distribution of lipopigments

**Figure 1:**
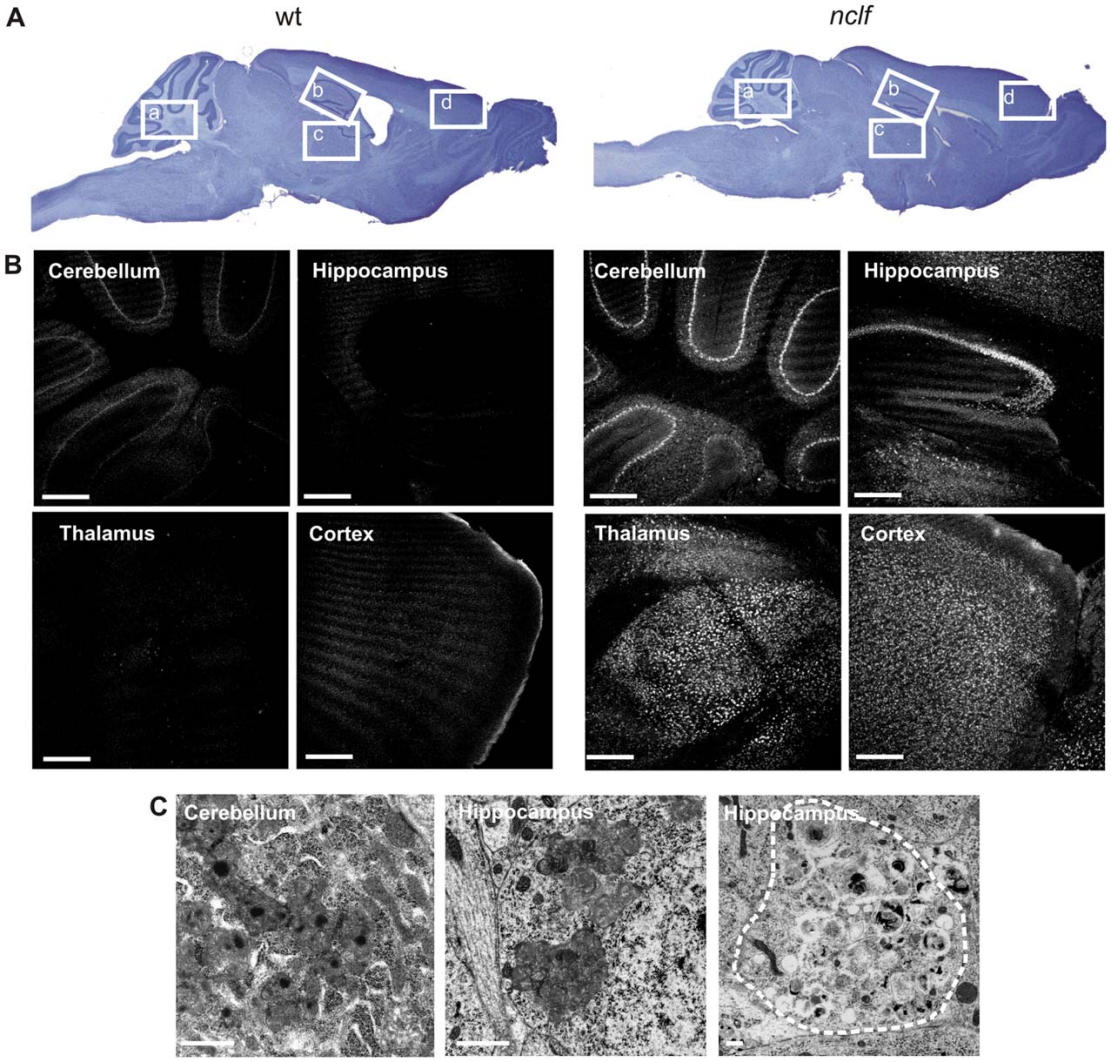
Storage material in *nclf* mouse brain. A) Sagittal mouse brain sections were stained with Nissl staining, showing cerebellar atrophy in the *nclf* brain. B) Autofluorescent storage material was evident in cerebellum, thalamus, hippocampus and cortex of 54 weeks old *nclf* mice but not in age-matched wild-type controls. The selected areas are shown in panel A (a, b, c, d). Scale bars: 300 µm. C) Ultrastructural analysis showed storage material in cerebellum and hippocampus of the brain of 52 weeks old *nclf* mice. Scale bar: 2 µm.

Examination of Nissl stained sagittal sections from 54 weeks old wild-type and *nclf* mice (Figure 1A) revealed reduced size of the cerebellum in *nclf* mice in comparison to controls. The median weight of *nclf* mice brain at one year of age was reduced by 25% compared to wild-type brains, indicating severe atrophy of the brain.

The distribution of the autofluorescent lysosomal storage material was evaluated on unstained sections of 54 weeks old *nclf* mice and age matched control animals by confocal fluorescence microscopy (Figure 1B). Lipopigments were found to accumulate throughout the entire brain in both neurons and microglia. In the cerebellum, Purkinje cells were the cell type with the greatest storage burden, bearing brightly autofluorescent storage material in the entire soma. The thalamus was populated with lipopigment-laden cells. All hippocampal neurons of Ammon's horn exhibited a bright autofluorescence. Neurons from the cerebral cortex contained graded levels of storage material with increasing amounts of lipopigments from layer I to layer V. Several other regions of the brain contained prominent accumulation of storage material, including the olfactory bulb. Ultrastructural examination of intralysosomal storage material (Figure 1C) revealed abnormally increased amounts of dense bodies with a typical lipopigment-like appearance with membrane bound rectilinear and fingerprint-profiles. Occasionally, *nclf* neurons exhibited swollen processes (Figure 1C) filled with heterogenous membrane bound storage vacuoles, dense and multilamellar bodies.

### Neuroinflammation in *nclf* mice

**Figure 2:**
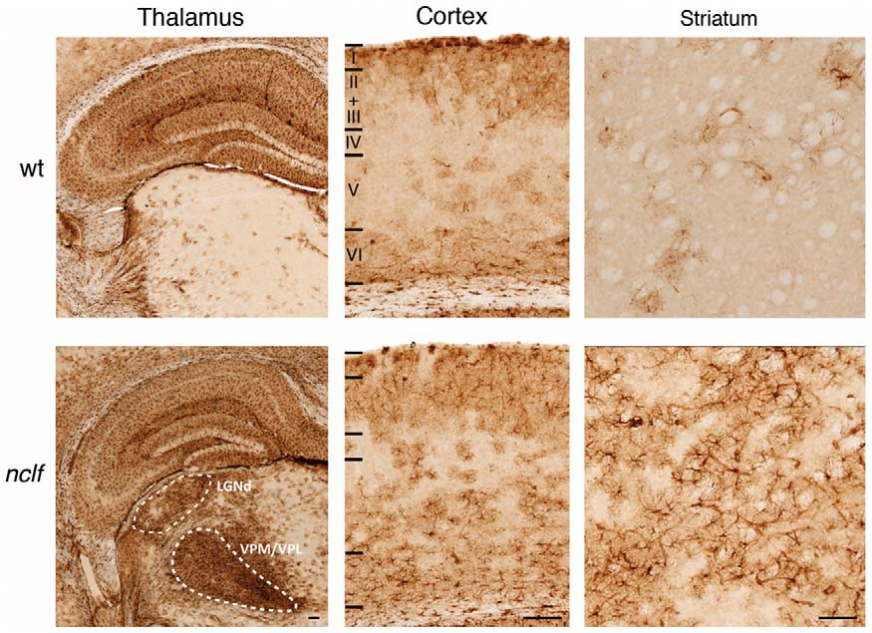
Localized astrocytosis in *nclf* mice. Immunohistochemical staining for glial fibrillary associated protein (GFAP) revealed pronounced upregulation of this marker of astrocytosis in 21 weeks old *nclf* mice compared to age-matched wild-type controls (wt). Intense localized astrocytosis was evident in the ventral posterior (VPM/VPL) and dorsal lateral geniculate (LGNd) relay nuclei of the thalamus of *nclf* mice, with more diffuse scattered GFAP positive astrocytes present predominantly in deeper (V-VI) and more superficial (I-III) laminae of the cortical mantle. Compared to wt controls, many GFAP stained astrocytes were also evident in the caudate-putamen of *nclf* mice. Laminar boundaries are indicated by roman numerals and white dashed lines indicate the boundaries of thalamic relay nuclei. Scale bar: 100 µm.

Activated astrocytes are found early in disease progression in CLN6-deficient New Zealand South Hampshire sheep [14], with localized GFAP immunoreactivity accurately predicting the distribution of subsequent neuron loss. Analysis of GFAP immunoreactivity in *nclf* mice revealed a similar distribution as in affected sheep with localized astrocytosis in individual nuclei of the thalamus and deeper cortical layers in presymptomatic mice at 21 weeks of age. Many intensely stained GFAP positive astrocytes were present in a variety of brain regions. As in mouse models of other forms of NCL [31], this localized astrocytosis was most prominent in individual thalamic sensory relay nuclei (Figure 2) including the ventral posterior (somatosensory relay) and dorsal lateral geniculate (visual relay) and the corresponding regions of somatosensory and visual cortex, together with the caudate putamen. In the cerebellum, astrocyte activation appeared relatively late (52 weeks) in disease progression and was restricted to the granular layer and white matter (Figure S1). No obvious loss of Purkinje cells was observed (data not shown), although these cells were particularly strong affected by accumulation of lipopigments (Figure 1B).

**Figure 3:**
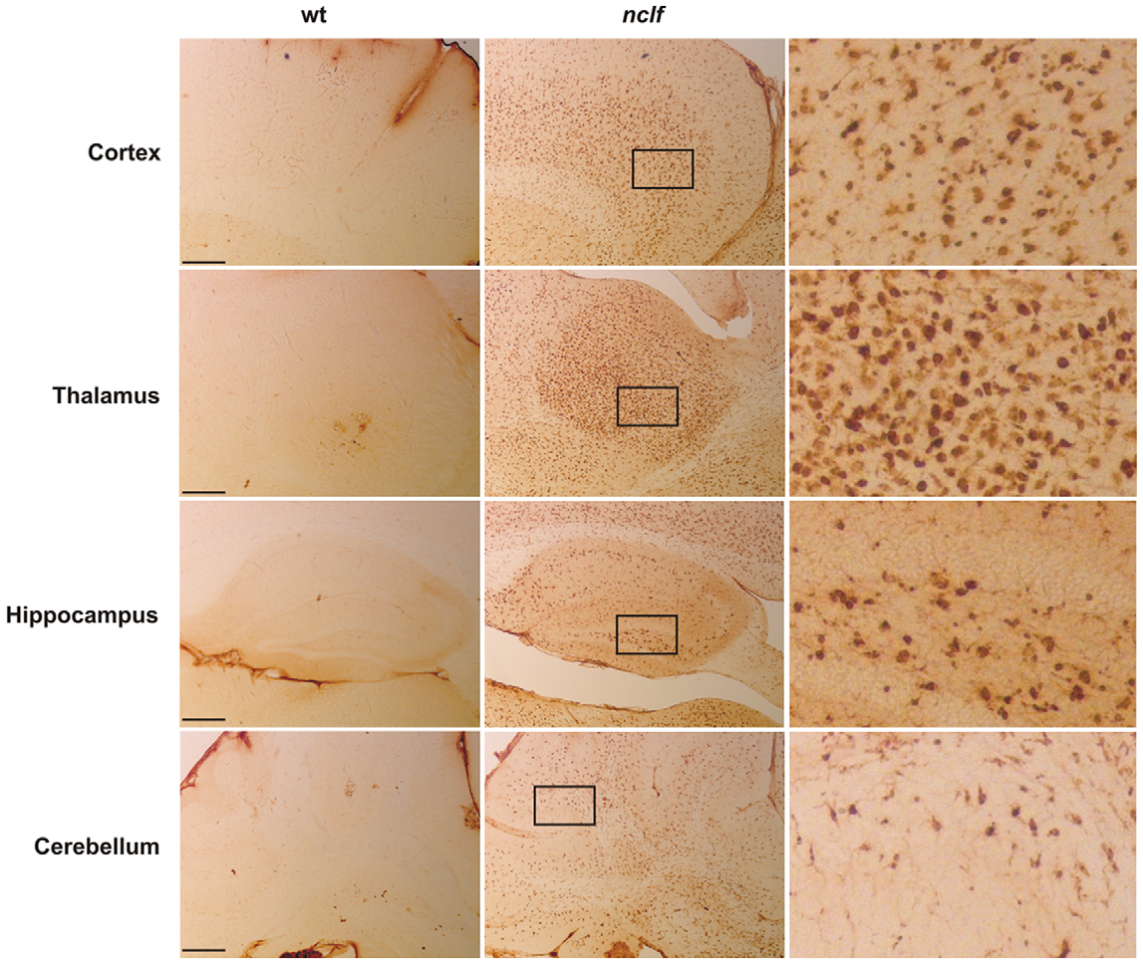
Activation of microglia in *nclf* brain. Immunohistochemical stainings of sagittal mouse brain sections showed a prominent microgliosis as assessed by the microglial marker CD68 at 54 weeks of age in *nclf* and wild-type (wt) mice. Scale bars. 500 µm. In the right panel, higher magnification images of the areas marked by the black rectangles are shown.

In addition to astrocytosis, microglial activation is common in other NCLs and a remarkable feature of CLN6-deficient sheep [14]. Highly activated microglia appeared early in the course of the disease of *nclf* mice, transforming to a phagocytotic shape in 54 weeks old animals (Figure 3). The distribution of activated microglia closely resembled that of lipopigment storage. Although local distribution of activated astrocytes and microglia was similar, with clusters of microglia in most nuclei of the thalamus, the olfactory bulb, caudate putamen, the granular layer and white matter of the cerebellum, microglial activation was more widespread and generalized in the entire brain. However, no prominent activation of perivascular macrophages was observed.

### Proteasomal degradation of mutant Cln6

**Figure 4:**
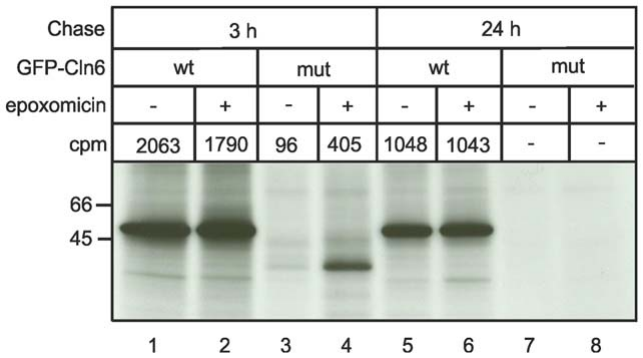
Mutant GFP-Cln6 is rapidly degraded by proteasomes. BHK cells overexpressing murine wild-type or mutant p.R103PfsX62 GFP-Cln6 (mut) were labelled for 24 hours with [^35^S]-methionine (75 µCi/ml) and either harvested or chased for 3 (lanes 1–4) and 24 hours (lanes 5–8) in the absence (–) or presence (+) of the proteasomal inhibitor epoxomicin (2 µM). GFP-Cln6 fusion proteins were immunoprecipitated, separated by SDS-PAGE (10% acrylamide) and revealed by fluorography. A representative experiment out of three is shown. The [^35^S]-labelled bands of the presented experiment were excised from the gel, solubilized and counted. The values are given above the lanes.

In order to investigate the potential role of the mutant Cln6 protein in the pathogenesis of the *nclf* mouse, we introduced the c.307insC mutation into the murine *Cln6* cDNA by site-directed mutagenesis. This insertion leads to a frame shift and a nonsense sequence of 62 amino acids, terminating in a premature stop codon (p.R103PfsX62). The synthesis and stability of the murine wild-type and mutant p.R103PfsX62 Cln6 were investigated in BHK cells overexpressing both proteins N-terminally fused to GFP. Transfected cells were metabolically labelled with [^35^S]-methionine for two hours and chased for different time periods followed by immunoprecipitation and fluorography. Wild-type GFP-Cln6 is synthesized as a 55 kDa protein. Quantification of radioactivity incorporated into immunoprecipitated GFP-Cln6 revealed a reduction of more than 50% during a 24 hours chase period (Figure 4, lanes 1 and 5). In contrast, small amounts of 42 kDa mutant p.R103PfsX62 GFP-Cln6 protein were synthesized and after 24 hours chase no [^35^S]-labelled polypeptides were recovered by immunoprecipitation (Figure 4, lanes 3 and 7). In the presence of the irreversible proteasomal inhibitor epoxomicin (2 µM) the amounts of immunoprecipitated wild-type GFP-Cln6 were not altered (Figure 4, lanes 2 and 6), whereas epoxomicin prevented the degradation of mutant p.R103PfsX62 GFP-Cln6 (Figure 4, lane 4). Under these conditions, however, epoxomicin did not prevent the degradation of mutant Cln6 during the 24 hours chase period (Figure 4, lane 8). Taken together, these data demonstrate that mutant p.R103PfsX62 Cln6 is synthesized as a truncated protein that is rapidly degraded by the proteasome system.

### ER stress is not activated in brains of *nclf* mice

**Figure 5:**
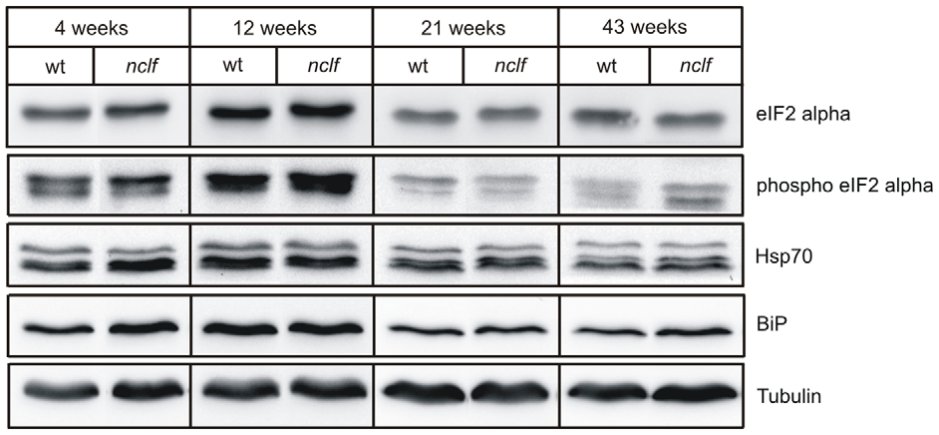
Analysis of ER stress and unfolded protein response markers in *nclf* mice. Cerebellar extracts from wild-type and *nclf* mice of 4, 12, 21, and 43 weeks of age were analyzed for eIF2alpha phosphorylation and for Hsp70 and BiP expression by western blotting. No major differences were detectable between the genotypes. Tubulin was used as loading control. Representative blots of three mice of different litters are shown.

To examine whether the rapid proteasomal degradation of mutant p.R103PfsX62 Cln6 leads to ER stress and the activation of UPR, the expression of several ER stress marker proteins was examined in cerebellar extracts of 4, 12, 21 and 43 weeks old *nclf* mice. Densitometric evaluation of western blots normalized to the tubulin expression revealed no significant differences in phosphorylation of eukaryotic initiation factor 2 alpha (phospho eIF2alpha) and expression of immunoglobulin heavy chain binding protein BiP and cytosolic heat shock protein 70 (Hsp70) between wild-type and *nclf* mice of any age (Figure 5). Similar results were obtained when extracts of cerebral cortex were analyzed (data not shown). Immunohistochemical staining failed to show regional or temporal differences in the expression of BiP and Hsp70 between wild-type and *nclf* mice (data not shown). These data suggest that the proteasomal degradation of mutant p.R103PfsX62 Cln6 is sufficient to prevent the accumulation of misfolded Cln6 protein and the subsequent activation of ER stress.

### Increased autophagy in the *nclf* mouse brain

**Figure 6:**
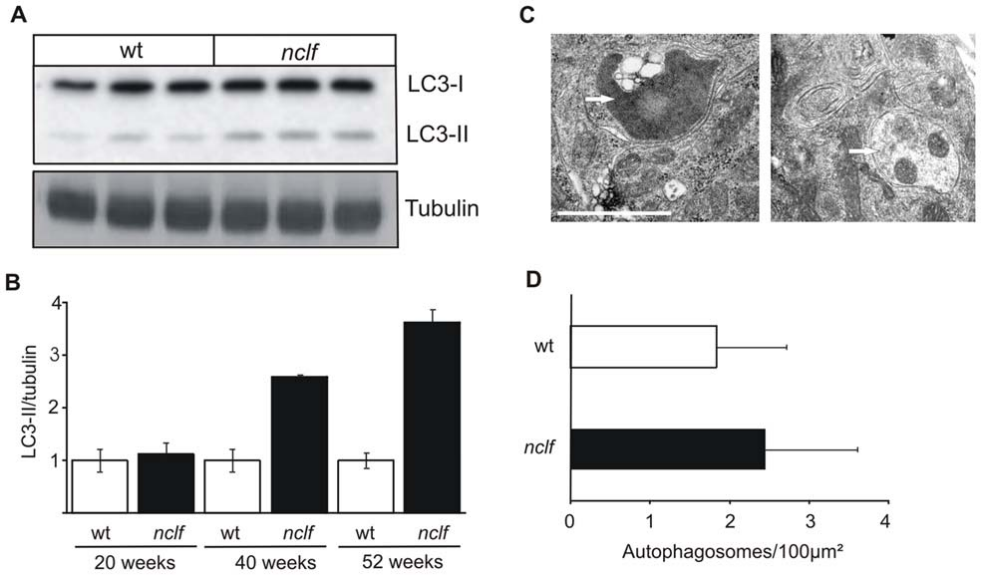
Accumulation of autophagosomes in *nclf* brain and hippocampal neurons. A) Accumulation of autophagosomes was assessed by determining the levels of the autophagic marker protein microtubule-associated protein 1 light chain 3 -II (LC3-II) in wild-type or *nclf* total mouse brain extracts at 54 weeks of age by western blotting. B) Densitometric quantification of LC3-II levels normalized to tubulin as a loading control revealed enhanced autophagosome numbers with increasing age. Data are presented as mean ±SD, n = 3 per age. Wild-type values were set to 1. C) Double-membrane autophagic vacuoles (marked by arrows) were also found in hippocampal neurons from *nclf* mice cultured for 14 days. D) For quantification of autophagic vacuoles, pictures were taken from 37 randomly selected wild-type and *nclf* neurons of two different preparations. The number of autophagic vacuoles related to the cytoplasmic area was determined. Data are presented as mean ± S.E.M. Scale bars: 1 µm.

Since autophagy has been shown to play a role in the pathogenesis of other forms of NCL, including CLN3 and CLN10 [5], [28], [29], we investigated whether this lysosomal dysfunction in *nclf* mice is also evident in CLN6 disease. The lipidated autophagosomal marker microtubule-associated protein 1 light chain 3-II (LC3-II) and p62 are known to accumulate upon inhibition of autophagy [25]. Comparative LC3 western blots of whole brain extracts of wild-type and *nclf* mice at different ages revealed 2.6- and 3.6-fold higher levels of LC3-II in 40 and 52 weeks old *nclf* mice, respectively (Figure 6 A, B). In presymptomatic 20 weeks old mice, no significant differences in the LC3-II levels could be detected between wild-type and *nclf* mice (Figure 6 B). These results could be verified by LC3 immunohistochemistry (Figure S2 B), showing increased amounts of granular LC3 positive material in the brains of older *nclf* mice. A small, but not statistically significant, increase in the number of autophagic vacuoles could also be observed in hippocampal neurons of *nclf* mice cultured for 14 days (Figure 6 C, D).

**Figure 7:**
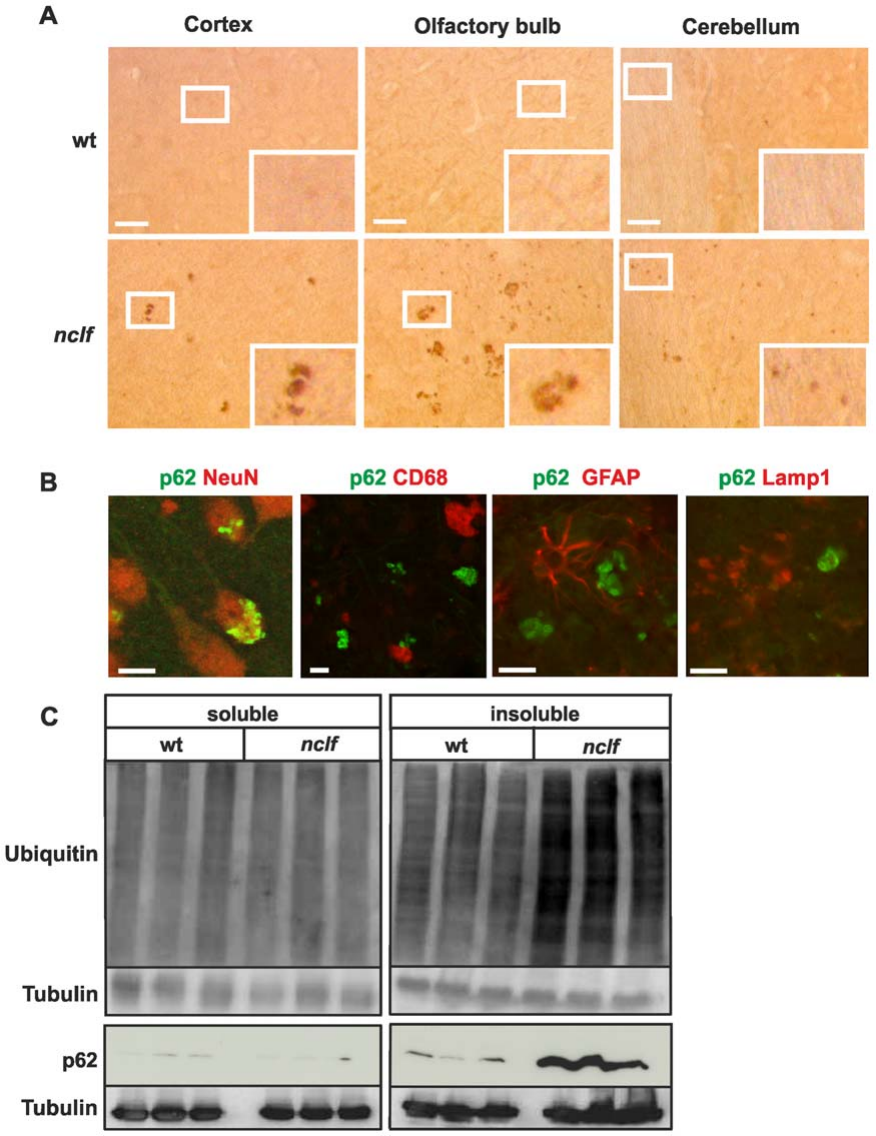
Aggregates of p62 and ubiquitinated proteins in brains of *nclf* mice. A) Immunohistochemical analysis of brain sections (35 µm thickness) of 54 weeks old mouse showed p62-positive aggregates in *nclf* but not wild-type brain regions. The insets show higher magnification images of the areas marked by the white rectangles. Scale bar: 20 µm. B) p62-positive accumulations showed no colocalization with microglial marker CD68, astrocytic marker GFAP or the lysosomal membrane protein Lamp-1 as determined by immunofluorescence microscopy in sections of the olfactory bulb in 54 weeks old *nclf* mice. Scale bar: 20 µm. C) Western blot analysis confirmed increased p62 levels and show furthermore accumulation of ubiquitinated proteins in Triton X-100 insoluble fractions of *nclf* brain.

p62 has been reported to act as a receptor for autophagic cargo by interaction with ubiquitinated proteins and LC3 [32]. In brains of *nclf* mice, p62-positive material could be detected already at an age of 20 weeks (Figure S2 A) in neurons of the olfactory bulb, cerebral cortex and the hippocampus. In 54 weeks old *nclf* mice (Figure 7 A), numerous p62-positive aggregates where found in the entire brain, but less frequently in the cerebellum and medulla. The size of p62-positive aggregates varied considerably between 1 and 10 µm. The distribution of p62 aggregates only partially resembled that of lipopigment material. In the thalamus, which showed a prominent accumulation of lipopigments (Figure 1 B), no p62-positive aggregates were found. In contrast, in layer I of the cortex, that exhibited high levels of p62 aggregates, lipopigments were absent. Double immunofluorescence microscopy showed a colocalization of p62 with NeuN-positive neuronal cells, but not with glial cells stained for the microglial and astrocytic markers CD68 and GFAP, respectively (Figure 7 B). To examine whether the increase in the number of LC3-II and p62-positive autophagosomes is due to signalling events initiating autophagy [25], the expression of Beclin-1 was determined in brain extracts of *nclf* mice at different ages. No changes in the expression of Beclin-1 were observed in the brain between wild-type and *nclf* mice (Figure S3). Since p62 did not colocalize with the lysosomal marker protein Lamp1 (Figure 7 B), the data suggest that the fusion between p62-positive autophagosomes and lysosomes is inhibited.

Western blot analysis showed that p62 was mainly found in the Triton X-100 insoluble fraction of *nclf* brain extracts, whereas only low amounts of p62 were observed in wild-type brain (Figure 7 C). In addition, the p62-positive insoluble fraction of *nclf* brain contained high levels of ubiquitinated proteins (Figure 7 C). These data suggest that the lysosomal dysfunction in neurons of *nclf* mice leads to accumulation of autophagosomes, most likely due to defective fusion processes with lysosomes and subsequent formation of aggregates of p62 and ubiquitinated substrate proteins.

## Discussion

The *nclf* mouse provides a valuable tool for investigating the molecular mechanisms underlying this variant form of late infantile NCL. The single cytosine insertion (c.307insC) in the *Cln6* gene of *nclf* mice was also found in three families of Pakistan origin (c.316insC). These mutations are responsible for frameshifts and production of truncated proteins, p.R103PfsX62 and p.R106PfsX26, respectively [8], [9], [12]. The present expression studies showed that the newly synthesized mutant p.R103PfsX62 Cln6 is rapidly degraded similarly to mutant human CLN6 [12]. The comparable half-lives of mutant human CLN6 and murine Cln6 indicate that the GFP-tag does not affect the stability of the mutant Cln6 protein. Inhibitor studies suggest that both mouse and human truncated Cln6/CLN6 proteins are degraded after retranslocation to the cytoplasm by the ubiquitin-proteasome pathway. Due to the lack of sufficiently specific antibodies recognizing the endogenous murine Cln6 protein, it remains unclear whether the proteasomal degradation is sufficient to prevent i) the accumulation of the misfolded mutant p.R103PfsX62 Cln6 in the ER or ii) its aggregation in the cytosol.

However, the accumulation of misfolded mutant Cln6 in ER of *nclf* brain cells and the subsequent activation of ER stress and UPR is rather unlikely, because we observed a rapid degradation of mutant Cln6 and we did not find changes in the expression of BiP, an ER luminal chaperone and marker of UPR [33]. Therefore, our data do not support the assumption that UPR represents a common pathway activated in neurodegenerative lysosomal storage diseases [21]. In line with our data, Futerman and colleagues failed to find evidence of activated UPR in models of neuropathic Gaucher disease [34]. Therefore, whereas activation of UPR has been reported in brain tissue and cultured neurons of mice deficient for the lysosomal palmitoyl protein thioesterase-1, a mouse model of human infantile NCL (CLN1) [22], our data lead us to conclude that different pathogenetic mechanisms are responsible for the relatively similar clinical symptoms and neurodegeneration in *Cln1*-deficient and *Cln6*-defective mouse models.

The cytosolic aggregation of mutant p.R103P*fs*X62 Cln6 also appears to be unlikely, because no alterations in the expression of Hsp70 were observed in brain extracts of *nclf* mice. Hsp70 plays an important role in the retranslocation and clearance of misfolded polytopic membrane proteins [35]. Another pathway for the constitutive clearance of damaged organelles and aggregated proteins that might be toxic, especially in post-mitotic cells like neurons, is autophagy [36]. Autophagy can be rapidly induced, e.g. by nutrient starvation or UPR, or can operate constitutively to degrade long-lived or aggregate-prone proteins, culminating in the formation of double membrane autophagosomes [25]. This process is assisted by the formation of a lipidated autophagosome-specific protein, LC3-II, whose content correlates with the number of autophagosomes [37]. The segregated cytosolic material and organelles, such as mitochondria, are then degraded by lysosomal hydrolases after fusion with lysosomes. Whereas the majority of degradation products are exported by membrane transporters to allow their reuse, indigestible material and lipopigments may remain in autolysosomes. Autophagy has been reported to be blocked during the pathogenesis of several lysosomal storage diseases, including CLN3 and CLN10 [28], [29], [38]. The presence of autophagic vacuoles in cultured neuronal cells and in the brain of *nclf* mice, and the age-dependent increase in LC3-II immunoreactive material suggests that the autophagic degradative pathway is upregulated or impaired. Impairment of autophagosome-lysosome fusion might be due to accumulation of undegraded substrates and/or lysosomal dysfunction [38], [39]. Although the origin(s) of the membranes that form new autophagosomes are unclear, it has been proposed that the ER supplies the material for the growing membrane [30]. Thus, disruption of ER function caused by mutant Cln6 could interfere with the assembly of the autophagosomal membrane and subsequently block this pathway.

Our electron microscopic analysis revealed that intralysosomal storage material was already present at postnatal day 10, increasing with age in *nclf* mice. p62-positive, detergent-insoluble material and p62-positive neuronal aggregates have been found in the brains of presymptomatic *nclf* mice. The multifunctional LC3-II and ubiquitin-binding protein p62 [32] mediates the degradation of ubiquitinated proteins via the autophagosome-lysosome pathway. Therefore, the reduced autophagic activity in the *nclf* brain is accompanied by the accumulation of ubiquitinated proteins in p62-positive cytoplasmic aggregates known to be toxic for cells. A coordinated and/or compensatory action of the ubiquitin-proteasome degradative pathway and autophagy has already been described for other proteins, including alpha-synuclein and alpha1-antitrypsin Z [40], [41], [42].

A consistent feature of NCL pathogenesis is the early and localized activation of astrocytes [18]. This phenotype was first described in CLN6-deficient South Hampshire sheep [14], and our data from *nclf* mice reveal for the first time that this is maintained across species boundaries, being present in this mouse model of CLN6 disease. Although early astrocytosis invariably precedes neuron loss in multiple forms of NCL [31], [43], [44], it is presently unclear whether these events are causally related in CLN6 deficiency. As in many other forms of NCL, it is clear that the thalamocortical system is a particular focus of astrocyte activation in *nclf* mice, but to date very little is known about the sequence of neuron loss in these pathways. Since it is becoming clear that the precise nature and relative timing of astrocytosis, microglial activation and neuron loss differ markedly between forms of NCL [45], it will be important to define the onset and progression of these events more precisely in *nclf* mice. Although these findings contribute to the understanding of the complex processes and mechanisms of progressive neurodegeneration in *nclf* mice as a model of the human CLN6 disease, the function of the ER-resident CLN6 protein and how mutant CLN6 leads to lysosomal dysfunction require further studies.

## Materials and Methods

### Materials

Dulbecco's Modified Eagle's Medium (DMEM), Minimum Essential Medium (MEM), fetal calf serum (FCS), Lipofectamine 2000, Taq polymerase and pcDNA3.1/myc-His(+)A vectors were purchased from Invitrogen (Karlsruhe, Germany). pEGFP-C1 vector was from Clontech (Mountain View, USA). [^35^S]-methionine and Rainbow molecular weight marker were obtained from GE Healthcare Europe (Freiburg, Germany), epoxomicin from Calbiochem (Darmstadt, Germany), PhosSTOP phosphatase inhibitor cocktail was purchased from Roche (Mannheim, Germany), Phusion® High-Fidelity DNA polymerase from Finnzymes (Espoo, Finland) and Protran® nitrocellulose membrane from Whatman (Dassel, Germany). Protease inhibitor cocktail and poly-L-lysine were purchased from Sigma (Munich, Germany). Oligonucleotides used for cloning and sequencing were synthesized by Eurofins MWG Operon (Ebersberg, Germany).

### DNA constructs

Murine *Cln6* cDNA (NM_001033175) was purchased from Deutsches Ressourcenzentrum für Genomforschung RZPD (Berlin, Germany), amplified by PCR using primers 5′-GAATTCATGGAGGCGGCGACGCG-3′ and 5′-CTCGAGCTGTTGACTGCTAACGTG-3′ and the resulting PCR product cloned into the *Eco*R I- and *Xho* I-sites of expression vector pcDNA3.1/myc-His(+)A. The c.307insC mutation was introduced into this murine *Cln6* cDNA using the QuickChange™ Site-Directed Mutagenesis Kit (Stratagene, La Jolla, CA, USA) according to the manufacturer's instructions using the following primers: 5′-CCGCGGCAGCGTTCGGGGGGGACCGC–3′ and 5′-GCGGTCCCCCCCGAACGCTGCCGCGG–3′. Murine wild-type and c.307insC Cln6 cDNA were cloned into pEGFP-C1 vector, downstream of the green fluorescent protein (GFP) cDNA sequence.

All constructs were sequenced using ABI PRISM BigDye Terminator Cycle Sequencing Kit on a Model 377 automated sequencer (Applied Biosystems, Darmstadt, Germany).

### Antibodies

Monoclonal anti-eIF2alpha, anti-phospho-eIF2alpha and anti-ubiquitin (clone P4D1) antibodies were purchased from Cell Signaling Technology (Danvers, MA, USA), monoclonal anti-Hsp70 and anti-BiP from BD Biosciences (Heidelberg, Germany), monoclonal anti-GFP antibody from Roche, monoclonal anti-LC3 antibody (clone 2G6) was from nanotools Antikörpertechnik (Teningen, Germany). Monoclonal mouse and polyclonal rabbit anti-GFAP antibodies were purchased from Sigma-Aldrich (St Louis, MO, USA) or DakoCytomation (Glostrup, Denmark), respectively. The monoclonal mouse anti-neuronal nuclei (NeuN) antibody (clone A60) was obtained from Millipore (Billerica, MA, USA). Monoclonal rat anti-CD68 antibody (clone FA-11) was purchased from AbD Serotec (Oxford, UK). Polyclonal rabbit anti-p62 antibody was purchased from ENZO Life Sciences (Plymouth Meeting, PA, USA) (immunohistochemistry) or MBL International (Woburn, MA, USA) (western blotting). The anti-beta tubulin monoclonal antibody (clone E7) developed by M. Klymkowsky was obtained from the Developmental Studies Hybridoma Bank developed under the auspices of the NICHD and maintained by The University of Iowa, Department of Biological Sciences, Iowa City. Horseradish peroxidase-conjugated goat anti-mouse and goat anti-rabbit IgG were obtained from Dianova (Hamburg, Germany). Alexa Fluor-conjugated secondary antibodies were obtained from Molecular Probes (Eugene, Oregon, USA). Biotinylated-secondary antibodies were purchased from Vector Laboratories (Burlingame, CA, USA).

### Animals

Cln6-defective *nclf* mice (B6.Cg-Cln6*^nclf^*) were purchased from The Jackson Laboratory (Bar Harbor, ME, USA). Animals were maintained according to the institutional guidelines of the animal facility of the University Medical Center Hamburg-Eppendorf. Killing and removal of brains from mice were approved by the local animal welfare officer, Dr. A. Haemisch (#ORG 532). Tissues were either immediately frozen and kept at −80°C prior analysis or used directly for analytical experiments.

### Cell cultures and transfections

Baby hamster kidney 21 (BHK) cells were kindly provided by Dr. K. von Figura (University of Göttingen; [46]) and grown in DMEM supplemented with 10% FCS at 37°C, 5% CO_2_, transiently transfected with Lipofectamine 2000 according to the manufacturer's instructions and further processed 18 h post-transfection. Hippocampal neurons from C57Bl/6J and *nclf* mice were prepared on postnatal day 0 as previously described [47] and plated at a density of 150 cells/mm^2^ on poly-L-lysine coated vessels.

### Metabolic labelling and immunoprecipitation

BHK cells expressing murine wild-type or mutant GFP-Cln6 were starved for 1 h in methionine-free MEM, labelled for 2 h with [^35^S]-methionine (75 µCi/ml) and either harvested or chased in DMEM supplemented with 10% FCS and methionine (5 mM) for up to 24 h. Where indicated, proteasome inhibitor epoxomicin (2 µM) was added during the pulse and chase periods. Cells were then lysed in PBS containing 1% Triton-X100, 1% bovine serum albumin and protease inhibitor cocktail followed by immunoprecipitation of GFP-Cln6 fusion proteins with anti-GFP antibody (5 mg/0.8 ml) and protein A agarose (Sigma). After washing, immunoprecipitates were solubilized and separated by SDS–PAGE under reducing conditions followed by fluorography. The radiolabeled polypeptides were quantified by excision of the bands from the gel, solubilization in Solvable tissue solubilizer (Perkin Elmer, Rodgau, Germany) and scintillation counting using a liquid scintillation analyzer Tri-Carb 2900TR (Perkin Elmer).

### Histological and microscopical analysis

For electron microscopic analysis, brains were dissected from C57Bl/6J and *nclf* mice at different ages, fixed with 4% paraformaldehyde and 1% glutaraldehyde and embedded in epoxy resin. Hippocampal neurons at DIV14 were fixed 30 min with 3% glutaraldehyde on the culture plates, collected by scraping, further fixed over night as a pellet with 3% glutaraldehyde and embedded in epoxy resin. Quantification of autophagosomes was performed as previously described [48]. Pictures were taken from randomly selected cells from each sample. For each picture, the number of autophagosomes and the total cellular area were determined and the number of autophagic structures per cell cytoplasmic area was calculated.

For GFAP-histological analysis brains were immersion fixed in 4% paraformaldehyde in 0.1 M phosphate buffer and 40 µm sections were cut and processed for immunohistochemical staining of free floating sections as described previously [48], using sequential incubation in polyclonal rabbit anti-GFAP (1∶5000), biotinylated goat anti-rabbit (1∶1000), Vectastain Elite ABC reagent (1∶1000) and immunohistochemistry revealed by development with DAB (see above).

Immunohistochemistry for p62, CD68 and GFAP staining was performed essentially as described previously [49], [50]. Mice were anaesthetized and perfused with PBS followed by 4% paraformaldehyde (PFA) (w/v) in PBS, pH 7.4. Brains were removed, postfixed in the same fixative over night and subsequently cryoprotected by incubation in 30% (w/v) sucrose in PBS over night. Free floating 35 µm thick slices were sagittally sectioned using a Leica 9000s sliding microtome. Sections for IHC were washed two times with PBS and subsequently blocked with blocking solution (1% BSA (w/v), 4% (v/v) normal goat serum, 0.02% (w/v) saponin in PBS). Antibodies were appropriately diluted in antibody diluent solution (1% BSA (w/v), 1.5% (v/v) normal goat serum, 0.02% (w/v) saponin in PBS) and sections incubated over night at 4°C with primary antibodies. After washing four times with washing solution (0.02% (w/v) saponin in PBS), sections were incubated with biotinylated secondary antibodies (diluted in antibody diluent solution) for one hour at room temperature. After another four washes with washing solution, the sections were incubated with Vectastain ABC Elite solution (Vector Laboratories, Burlingame, CA, USA) for 1.5 hours at room temperature. After the last four washes, sections were developed using DAB substrate solution, mounted and coverslipped with Mowiol or DPX.

Double-immunofluorescence and autofluorescence were evaluated by confocal laserscan microscopy with a Leica Sp2 confocal microscope. Tissue sections were processed as described above except incubation for 1 hour with fluorophore-conjugated secondary antibodies (rabbit Alexa Fluor 488 and mouse/rat Alexa Fluor 633) instead of biotin-conjugated secondary antibodies. Sections were coverslipped subsequently in Mowiol containing DABCO (1,4-diazabicyclo(2,2,2)octan). For evaluation of autofluorescence, unstained sections were coverslipped as above and evaluated with the 488 nm laser line and emission at 519 nm ± 20 nm.

### Western blot

Tissue homogenates were prepared as follows: tissues (whole brain or cerebellum) were dissected from mice at each age and homogenized in either 1 ml (brain) or 0.5 ml (cerebellum) 20 mM Tris-HCl, pH 7.5, containing 150 mM NaCl, protease inhibitor cocktail and PhosSTOP phosphatase inhibitor cocktail using a dounce homogenizer. Proteins were solubilized by the addition of Triton X-100 to a final concentration of 1%. Extracts were cleared from insoluble material by ultracentrifugation for 1 h at 100,000×*g*, 4°C, using a Sorvall Discovery M120 centrifuge and protein concentration of the supernatant was determined with Bradford protein assay (Bio-Rad, Munich, Germany). Alternatively, for assessment of LC3 levels and fractionation of proteins, total brains were homogenized in five volumes of 250 mM sucrose buffer containing 50 mM Tris-HCl pH 7.4, 1 mM EDTA and protease inhibitor cocktail. Nuclei were cleared by centrifugation at 500×*g* for 10 minutes at 4°C and supernatants were lysed with an equal volume of sucrose buffer containing 1% Triton X-100 for one hour at 4°C. Resulting lysates were either used directly or further separated into Triton X-100 soluble/insoluble fractions by centrifugation at 13,000×*g* for 15 minutes at 4°C. Supernatants (representing the detergent-soluble fractions) were removed and pellets (representing the detergent-insoluble material) resuspended in 1% SDS in PBS with inhibitor cocktail.

Samples were separated by SDS-PAGE and blotted onto nitrocellulose membrane. Incubation of the primary antibody was followed by incubation with horseradish peroxidase-conjugated secondary antibody (1∶5,000) and visualization of the bands with enhanced chemiluminescence (ECL) and ChemiDoc XRS (Bio-Rad). Densitometric quantification was performed using the spot density function of ChemiDoc XRS.

## Supporting Information

Figure S1 **Astrocytosis in the cerebellum of** ***nclf*** **mice.** Immunohistochemical staining for GFAP in the cerebellum of 54 weeks old wild-type (wt) and *nclf* mice. Scale bars: 500 µm. In the lower panel, higher magnification images of the areas marked by the white rectangles are shown.
(TIF)

Figure S2 **Immunohistochemistry of p62 and LC3.** A) Immunohistochemical analysis of brain sections (35 µm thickness) of 20 and 54 weeks old mouse brain showed p62-positive aggregates in *nclf* brain regions. Scale bar: 20 µm. B) Immunohistochemical staining of LC3 in 54 weeks old wt and *nclf* hippocampus showed LC3-positive structures (arrows) in the *nclf* brain. Scale bar: 20 µm.
(TIF)

Figure S3 **No induction of autophagy by Beclin-1.** Beclin-1 expression was examined by western blotting of brain extracts of wild-type (wt) and *nclf* mice at different ages. Tubulin was used as loading control.
(TIF)
