# Correction: Disruption of the Autophagy-Lysosome Pathway Is Involved in Neuropathology of the *nclf* Mouse Model of Neuronal Ceroid Lipofuscinosis

**DOI:** 10.1371/annotation/a4b06d46-8eb9-4d15-a15a-41bf4b5ccb8b

**Published:** 2012-05-14

**Authors:** Melanie Thelen, Markus Damme, Michaela Schweizer, Christian Hagel, Andrew M.S. Wong, Jonathan D. Cooper, Thomas Braulke, Giovanna Galliciotti

There was a typographical error in the second author's name. The correct spelling is Markus Damme. The correct citation is: Thelen M, Damme M, Schweizer M, Hagel C, Wong AM, et al. (2012) Disruption of the Autophagy-Lysosome Pathway Is Involved in Neuropathology of the *nclf* Mouse Model of Neuronal Ceroid Lipofuscinosis. PLoS ONE 7(4): e35493. doi:10.1371/journal.pone.0035493 

