# Supplementary figures and images for: Disruption of the Autophagy-Lysosome Pathway Is Involved in Neuropathology of the nclf Mouse Model of Neuronal Ceroid Lipofuscinosis
Source: PLoS One. 2012 Apr 20;7(4):e35493. doi: 10.1371/journal.pone.0035493 (PMC3335005; doi:10.1371/journal.pone.0035493)

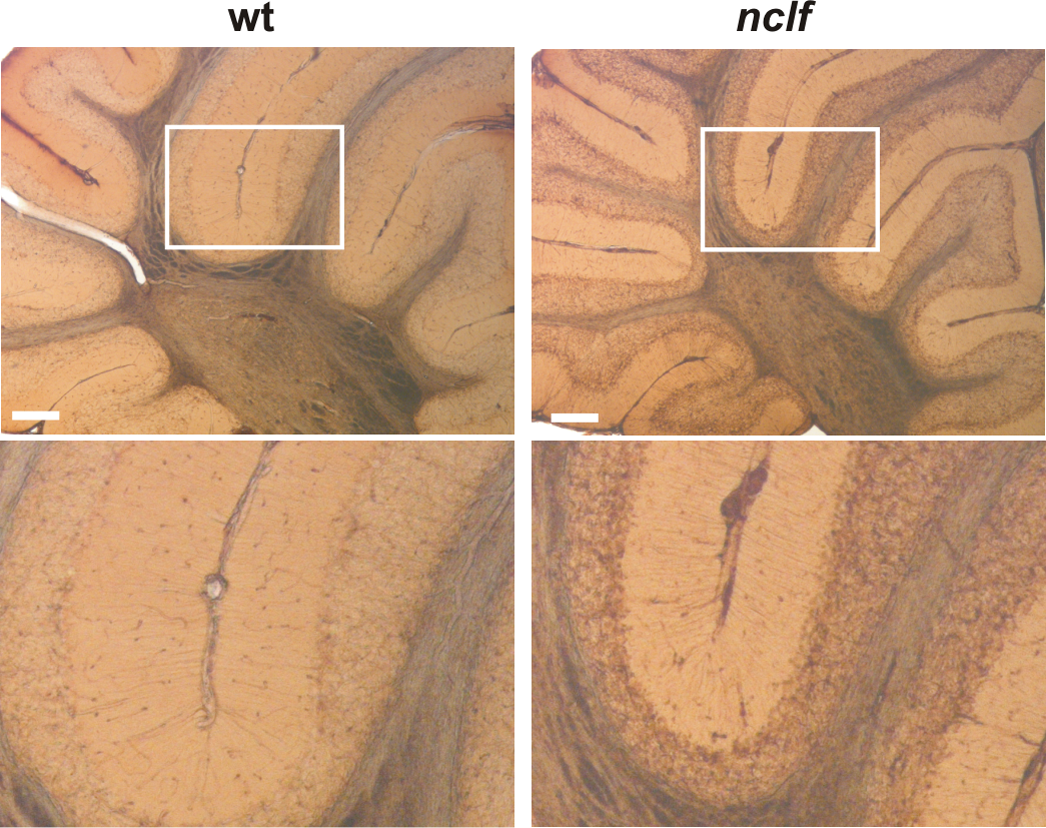

Supplement: Figure S1 — Astrocytosis in the cerebellum of nclf mice. Immunohistochemical staining for GFAP in the cerebellum of 54 weeks old wild-type (wt) and nclf mice. Scale bars: 500 µm. In the lower panel, higher magnification images of the areas marked by the white rectangles are shown. (TIF) [file pone.0035493.s001.tif]

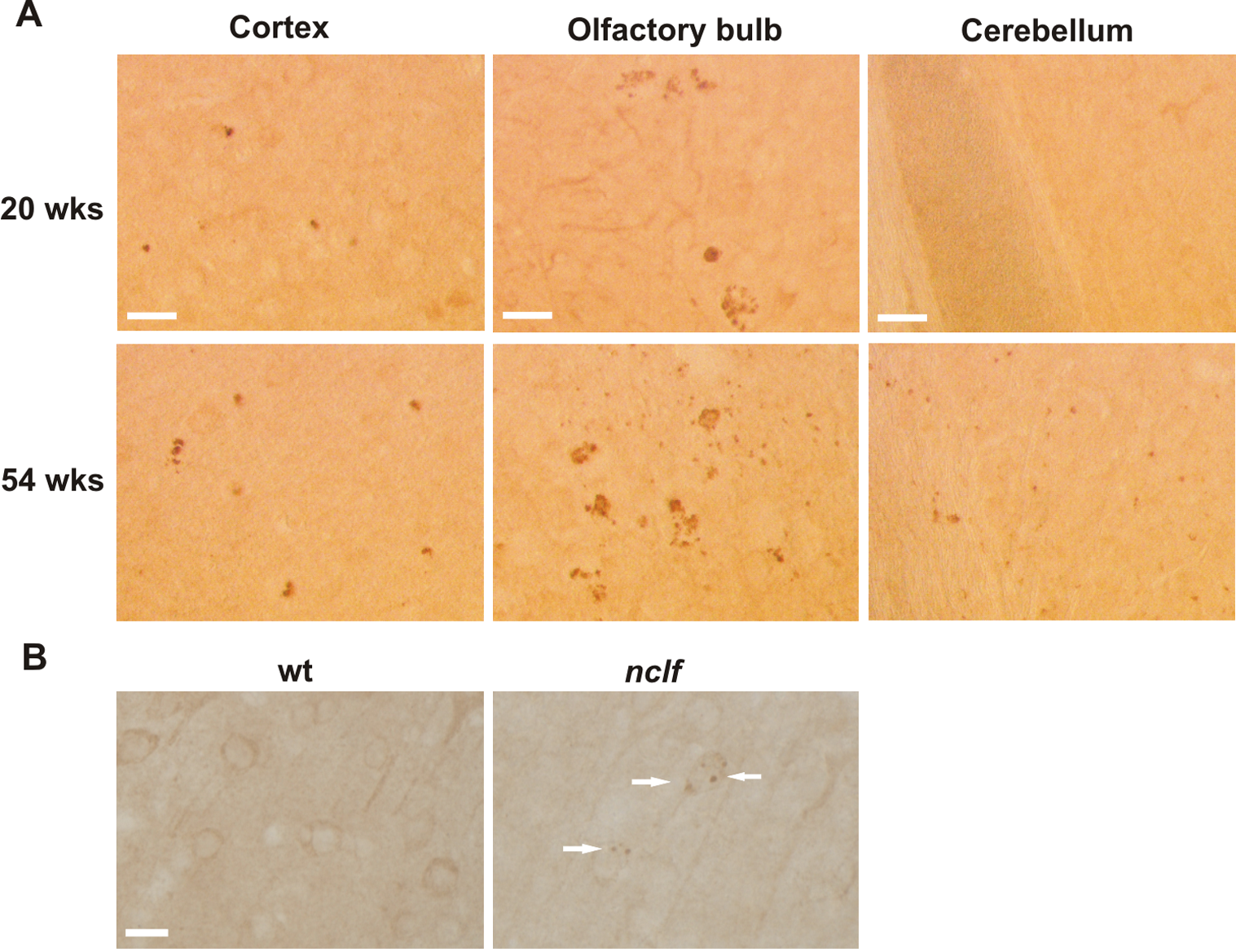

Supplement: Figure S2 — Immunohistochemistry of p62 and LC3. A) Immunohistochemical analysis of brain sections (35 µm thickness) of 20 and 54 weeks old mouse brain showed p62-positive aggregates in nclf brain regions. Scale bar: 20 µm. B) Immunohistochemical staining of LC3 in 54 weeks old wt and nclf hippocampus showed LC3-positive structures (arrows) in the nclf brain. Scale bar: 20 µm. (TIF) [file pone.0035493.s002.tif]

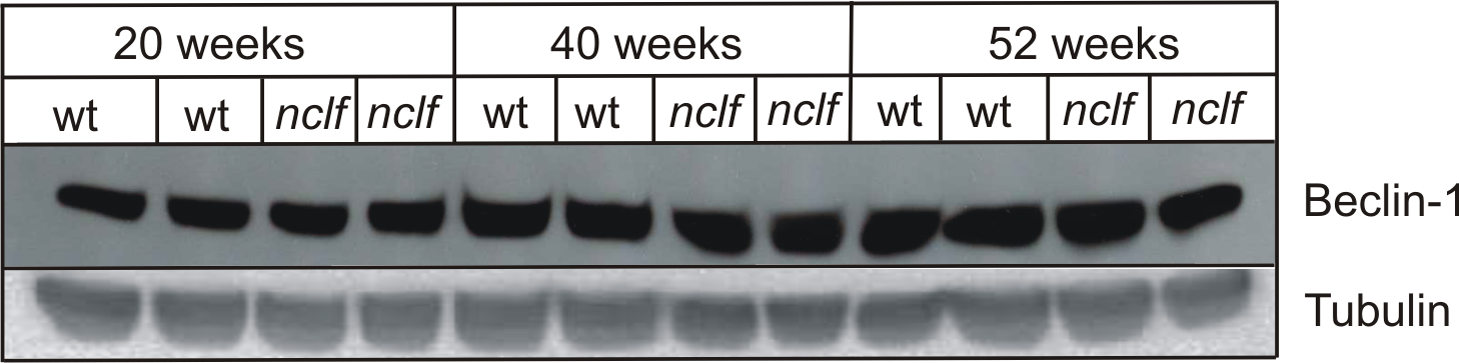

Supplement: Figure S3 — No induction of autophagy by Beclin-1. Beclin-1 expression was examined by western blotting of brain extracts of wild-type (wt) and nclf mice at different ages. Tubulin was used as loading control. (TIF) [file pone.0035493.s003.tif]
